# RNA Therapeutics for Duchenne Muscular Dystrophy: Exon Skipping, RNA Editing, and Translational Insights from Genome-Edited Microminipig Models

**DOI:** 10.3390/ijms27062755

**Published:** 2026-03-18

**Authors:** Alex Chassin, Hiroya Ono, Yuki Ashida, Michihiro Imamura, Yoshitsugu Aoki

**Affiliations:** 1 Department of Molecular Therapy, National Institute of Neuroscience, National Centre of Neurology and Psychiatry, Tokyo 187-8502, Japan; alex.chassin@etud.univ-evry.fr (A.C.); ashida.yuki@ncnp.go.jp (Y.A.); imamura@ncnp.go.jp (M.I.); 2 Tissular, Cellular and Gene Biotherapy, Université Paris-Saclay, 91190 Gif-sur-Yvette, France

**Keywords:** Duchenne muscular dystrophy, RNA therapeutics, antisense oligonucleotides, exon skipping, RNA editing, ADAR, phosphorodiamidate morpholino oligomer, peptide-conjugated PMO, antibody–oligonucleotide conjugate, microminipig, genome editing, animal models

## Abstract

Duchenne muscular dystrophy (DMD) is a severe X-linked neuromuscular disease (NMD) caused by loss-of-function mutations in the *DMD* gene. RNA-based therapies, especially antisense oligonucleotides (ASO)-mediated exon skipping and adenosine deaminase acting on RNA (ADAR)-guided RNA editing, have emerged as complementary approaches that modulate pre-mRNA splicing or correct transcripts without altering genomic DNA. Current phosphorodiamidate morpholino oligomer (PMO) drugs targeting exons 51, 53, and 45 provide mutation-class-specific benefit. At the same time, next-generation delivery strategies (e.g., peptide-conjugated PMOs (PPMOs), antibody–oligonucleotide conjugates (AOC), and endosomal-escape vehicles) aim to improve skeletal, cardiac, and diaphragm exposure. In parallel, RNA editing strategies offer a route to correct select nonsense or missense variants at the base level and may, in principle, restore near-native dystrophin expression. Meaningful translation of these modalities requires predictive large-animal models. A genome-edited microminipig (MMP) bearing *DMD* exon-23 mutations faithfully recapitulates hallmark features of human DMD. That includes early locomotor deficits, elevated serum creatine kinase (CK) and cardiac troponin T, progressive myocardial fibrosis, and a decline in left-ventricular ejection fraction (LVEF), while maintaining a manageable lifespan of approximately 30 months suitable for long-term studies. In particular, the MMP model provides a practical platform for addressing the persistent challenge of efficient therapeutic delivery to the heart and diaphragm through longitudinal dosing, imaging, and biopsy. In this review, we synthesize clinical progress in exon skipping, outline the promise of RNA editing, and integrate recent insights from Duchenne muscular dystrophy model for microminipigs (DMD-MMPs) as an advanced surrogate for preclinical development and translational evaluation.

## 1. Introduction

DMD is a severe, X-linked NMD that affects approximately 1 in 4000–5000 newborn boys worldwide each year, caused by mutations in the *DMD* gene, which encodes the dystrophin protein. This key structural component stabilizes the sarcolemma during muscle contraction [1]. The absence of functional dystrophin results in progressive muscle fiber degeneration, chronic inflammation, fibrosis, and premature death, typically due to respiratory or cardiac failure. Despite advances in supportive care, current treatments remain largely palliative, underscoring the urgent need for effective molecular therapies targeting the genetic root of the disease.

Beyond its mechanical role, the absence of dystrophin profoundly alters sarcolemmal integrity, leading to increased membrane permeability to ions, particularly Ca^2+^, but also Na^+^ and K^+^. This destabilization results in chronic Ca^2+^ influx, which activates calcium-dependent proteases such as calpains and phospholipases, promotes sarcoplasmic reticulum stress, and disrupts excitation–contraction coupling. This increase in sarcolemmal permeability represents the initiating event of a pathogenic cascade. Sustained Ca^2+^ entry acts as a central upstream trigger that sequentially activates calcium-dependent proteases, disrupts mitochondrial homeostasis, and amplifies oxidative stress. Sustained calcium overload further impairs mitochondrial homeostasis by enhancing reactive oxygen species (ROS) production, inducing mitochondrial membrane depolarization, opening of the mitochondrial permeability transition pore, and ATP depletion. Together, these events contribute to progressive myofiber damage, necrosis, and impaired regeneration [2,3].

At the tissue level, Ca^2+^ dysregulation and mitochondrial dysfunction act as potent triggers of chronic inflammation. Damaged myofibers release danger-associated molecular patterns that recruit and activate macrophages, while persistent injury promotes fibroblast activation and excessive extracellular matrix deposition. This inflammatory fibrotic cascade ultimately leads to irreversible muscle remodeling and progressive fibrosis in both skeletal and cardiac muscle, which underlies functional decline and cardiomyopathy in DMD [4,5].

DMD symptoms typically appear around age 2, including difficulty climbing stairs and standing up, and frequent falls. It causes delayed gross motor development, and most patients require a wheelchair by the age of 10–12. At the molecular level, dystrophin links cytoskeletal actin to the extracellular matrix, thereby protecting the sarcolemma from mechanical stress during muscle contraction [6]. Progressive muscle weakness leads to scoliosis and joint contractures, contributing to restrictive lung disease. Assisted ventilation is usually required between the ages of 15 and 20, and most patients die from cardiac or respiratory failure between the ages of 20 and 30 [7,8]. DMD can also affect cognitive function. Approximately 34.8% of patients are reported to have intellectual disabilities [9], including autism spectrum disorder, learning deficit and attention-deficit/hyperactivity disorder [10]. The identification of the *DMD* gene as central to DMD pathogenesis has led to a broader understanding of muscle membrane structure and the role of dystrophin in sarcolemmal stability. The *DMD* gene is the largest known gene in the human genome, spanning 2.5 megabases with 79 exons and 78 introns [11]. Due to its large size, the gene is susceptible to many types of mutations; over 7000 different mutations have been identified in DMD patients [12]. Mutations predominantly cluster in two regions of the *DMD* gene, located at exons 3–9 and 45–55, where deletions often result in frameshift mutations and loss of protein expression [13]. The *DMD* gene encodes multiple Dp isoforms. The 427 kDa full-length dystrophin protein, (Dp427), critical for sarcolemmal stability and expressed in skeletal and cardiac muscles, as well as in synaptic regions [14]. Shorter isoforms are also produced from internal promoters, such as Dp140 (from intron 44), and Dp71/Dp40 (from intron 62). These isoforms are predominantly expressed in the brain. Dp140 is present throughout the brain, while Dp71 and Dp40 are the most common dystrophin isoforms in the brain, expressed in the cerebellum and in the cerebral cortex [15,16]. Most mutations disrupt the open reading frame and generate premature termination codons. These aberrant transcripts are targeted by the nonsense-mediated decay pathway, leading to a near-total absence of functional dystrophin protein [17]. DMD represents the most well-characterized monogenic NMD and serves as a prototypical model for developing and evaluating RNA-based therapies. The genetic basis of DMD, caused by loss-of-function mutations in the *DMD* gene, makes it particularly amenable to exon-specific interventions, such as ASO-mediated exon skipping or RNA editing. In addition, the high disease burden, early onset, and unmet clinical needs justify strong research and regulatory focus on DMD compared to other NMD.

Without treatment, life expectancy for DMD patients is around 20 years [18]. Currently, glucocorticoids remain the standard pharmacological treatment. They significantly prolong ambulation and delay cardiac and respiratory complications, although they do not target the underlying genetic cause. Improvement in skeletal muscle function under corticosteroid therapy has been associated with preservation of cardiac and pulmonary capacity. Their widespread clinical use has delayed the onset of dilated cardiomyopathy, which remains the leading cause of death in DMD patients [14]. With this intervention, life expectancy has increased to around 40 years [19]. Nevertheless, glucocorticoids remain symptomatic treatments and do not correct the genetic defect. For this reason, innovative approaches are being developed, including RNA-based therapeutic strategies and oligonucleotide-based therapy to restore dystrophin expression in the DMD RNA [14]. Current therapeutic development for DMD encompasses diverse modalities, including exon skipping, stop-codon readthrough, adeno-associated viruses (AAV) gene replacement, and genome editing, yet RNA-based strategies remain among the most clinically advanced [20]. These emerging therapies may also address secondary neurocognitive symptoms associated with the disease.

In recent years, RNA-based strategies have emerged as among the most promising approaches for DMD, enabling modulation of gene expression or repair of defective transcripts without permanently altering genomic DNA. Among these, ASO-mediated exon skipping and ADAR-guided RNA editing have shown distinct yet complementary potential. Exon skipping aims to restore the dystrophin reading frame by inducing selective exon exclusion during pre-mRNA splicing, producing a truncated but partially functional dystrophin protein. Oligonucleotide-based therapy is a form of RNA-based therapeutic strategies that employs short synthetic strands of DNA, RNA or their chemical analogues to hybridize with specific RNA or DNA targets, leading to their inactivation [21]. The most developed therapeutic oligonucleotide is the ASO, which is a short, single-stranded DNA-like molecule of 15–30 nucleotides. ASO can hybridize RNA sequence motifs to modulate gene expression levels. They must undergo chemical modifications to acquire drug-like characteristics, including improved nuclease resistance, reducing the immune responses triggered by foreign DNA or RNA, and enhanced bioavailability [22]. In contrast, RNA editing enables correction of pathogenic mutations at the single-base level, potentially restoring full-length, native dystrophin expression. Together, these RNA-targeting modalities represent a rapidly evolving therapeutic frontier for DMD.

**Figure 1:**
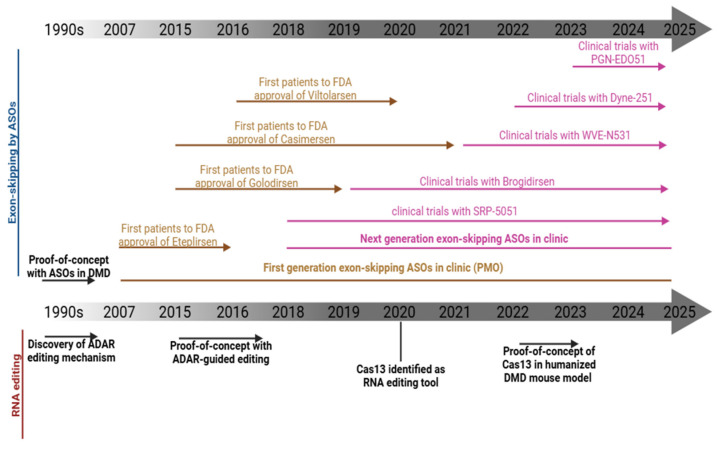
Timeline of exon skipping by ASO and RNA editing. ASO (antisense oligonucleotide); ADAR (adenosine deaminase acting on RNA); DMD (Duchenne muscular dystrophy); FDA (Food and Drug Administration); PMO (phosphorodiamidate morpholino oligomer).

The historical development and clinical progression of exon-skipping and RNA-editing approaches are summarized in Figure 1. These advances in RNA-targeting therapeutics, supported by substantial chemical and structural innovations, have markedly expanded the therapeutic landscape for DMD [23,24]. At the same time, their successful translation into clinical practice requires preclinical evaluation systems that can accurately predict therapeutic efficacy, biodistribution, and long-term safety under conditions relevant to human physiology.

While rodent models have been indispensable for elucidating molecular mechanisms and establishing proof-of-concept, their small body size and pronounced species differences in cardiovascular and skeletal muscle physiology limit their predictive value for systemically administered RNA therapeutics. In particular, the assessment of dose scalability, tissue distribution, repeated administration, and chronic safety profiles remains challenging in small-animal models. These limitations underscore the importance of large-animal models as a critical translational step bridging preclinical studies and human clinical trials [25].

Among available large-animal models, the MMP, the smallest miniature pig strain with an adult body weight of approximately 20 kg, offers distinct advantages for translational research in DMD [26]. Its body size is comparable to that of large-breed dogs, such as Golden Retrievers, enabling practical handling, procedural feasibility, and reduced dosing requirements for systemically delivered therapeutics. Moreover, the physiological and anatomical similarities between pigs and humans, particularly in cardiovascular and skeletal muscle systems, provide a strong foundation for clinically relevant evaluation [27]. These features allow large-animal studies to be conducted with improved feasibility and cost efficiency while retaining high translational relevance. Consequently, DMD-MMP model represents a crucial experimental platform that bridges the gap between rodent studies and human clinical application.

This review focuses on RNA-based therapeutic strategies for DMD, with particular emphasis on exon-skipping and RNA-editing technologies. In addition, the recently developed DMD-MMP model is discussed as a pivotal translational bridge for evaluating and optimizing these approaches. Over the past two decades, RNA-based strategies have evolved from single-modality interventions, such as exon skipping, into a diversified therapeutic platform that now includes RNA-editing tools. Exon skipping aims to restore the reading frame of dystrophin mRNA in patients with frame-disrupting mutations, whereas RNA editing enables precise base-level corrections without altering the genome. Together, these approaches constitute the most clinically advanced RNA-targeting strategies currently explored for the treatment of DMD. This review provides an overview of recent clinical advances in exon-skipping therapies and discusses emerging technologies, highlighting their current status, clinical relevance, and the challenges that remain before successful translation into routine care. Among these strategies, exon skipping has emerged as the most clinically advanced and extensively studied approach, forming the cornerstone of therapeutic development for DMD.

## 2. Exon Skipping Therapy for Duchenne Muscular Dystrophy

### 2.1. Principles and Applications of Exon Skipping

Exon skipping can spontaneously restore the dystrophin mRNA reading frame in patients with DMD, as well as in animal models. In dystrophic muscle tissue, discrete clusters of dystrophin-positive revertant fibers are frequently observed, and their number increases progressively with repeated cycles of degeneration and regeneration. The re-expression of dystrophin in these fibers is attributed to spontaneous exon skipping events that bypass the site of the primary mutation [14].

The most widely used application of ASO in DMD is steric-blocking, particularly through splice-switching approaches. Steric-blocking ASO binds to specific pre-mRNA sequence motifs and interferes with spliceosome recognition, thereby redirecting pre-mRNA splicing and restoring a translatable mRNA reading frame. In DMD, this strategy enables the production of a truncated but partially functional dystrophin protein, effectively converting a severe dystrophin-deficient phenotype into a milder Becker muscular dystrophy (BMD)-like phenotype [19,28]. BMD is an allelic variant of DMD caused by in-frame mutations of the *DMD* gene that preserve dystrophin expression, but at reduced levels [29].

### 2.2. Molecular Mechanisms of Exon Skipping

Exon skipping in RNA-based therapy is most commonly achieved using ASO. These short, synthetic single-stranded DNA or RNA analogues are designed to hybridize with specific pre-mRNA sequences and sterically interfere with spliceosome assembly, thereby modulating splicing or gene expression. Over the past decades, several generations of ASO have been developed to enhance their pharmacological and biochemical properties. The earliest ASOs were largely unmodified and highly susceptible to endonucleases and exonucleases, severely limiting their therapeutic potential. The first limitation was instability; to address it, a key modification introduced in the first generation was the substitution of a non-bridging phosphate oxygen with a sulfur atom, creating the phosphorothioate backbone and improving nuclease resistance and plasma protein binding. This modification enhanced their binding to plasma proteins and increased their resistance to nuclease activity, thereby prolonging their half-life [30]. The second-generation incorporated sugar modifications, such as 2′-O-methyl and 2′-O-methoxyethyl groups, often combined with a PS backbone. These modifications further improved nuclease stability and reduced immune activation. However, they generally exhibited lower affinity for their targets compared to other modified ASOs [22]. 2′-OMe ASO was among the first promising ASOs tested in clinical trials. The third generation, represented by PMO, replaced the ribose ring with a morpholine moiety and the charged phosphodiester linkage with a neutral phosphorodiamidate bond [31]. This configuration provides exceptional nuclease resistance and biostability but limits cellular uptake due to the absence of charge [32]. To overcome this limitation, PPMOs were developed, coupling cell-penetrating peptides (CPPs) to the PMO backbone. CPPs, composed of 5–30 amino acids rich in arginine or lysine, enhance cellular internalization via direct membrane penetration or endocytosis, thereby increasing exon-skipping efficacy in muscle tissues [33,34]. PPMOs exhibit an improved pharmacokinetic (PK) profile compared to PMOs [29].

**Figure 2:**
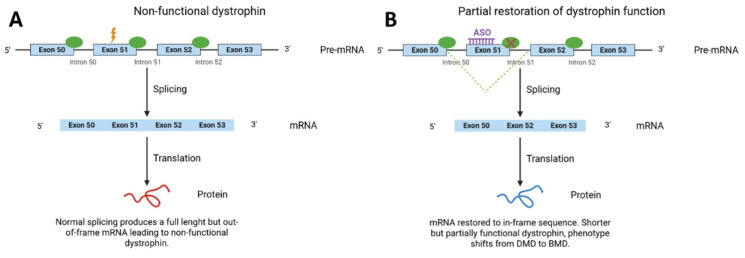
Mechanism of exon 51 skipping in DMD using PMO-based ASO. (**A**) In the absence of ASO treatment, exon 51 is included during pre-mRNA splicing, resulting in a frame-disrupted transcript. (**B**) Binding of a steric-blocking PMO to splice regulatory sequences prevents recognition of exon 51, leading to its exclusion from the mature transcript. Rectangles represent exons, solid lines represent introns, and circles represent spliceosome. ASO (antisense oligonucleotide); DMD (Duchenne muscular dystrophy); PMO (phosphorodiamidate morpholino oligomer); BMD (Becker muscular dystrophy).

ASO can regulate gene expression through two principal mechanisms: RNase H-dependent degradation and steric blocking (splice-switching). Steric-blocking ASO, such as those used for exon skipping in DMD, do not activate RNase H. Instead, they bind to pre-mRNA at splice junctions or regulatory splicing motifs, thereby preventing spliceosome assembly and exon recognition. This splicing interference alters exon recognition by the spliceosome and promotes selective exon exclusion during pre-mRNA processing. The molecular mechanism underlying ASO-mediated exon skipping is illustrated in Figure 2. Building on these mechanistic principles, splice-switching ASO have been successfully adapted for therapeutic use in DMD, where they are designed to mask splice sites or splicing enhancer motifs and promote exclusion of targeted exons [28,35].

Despite their therapeutic potential, unmodified PMOs exhibit significant limitations in cellular uptake. High doses are often required to achieve therapeutic efficacy, which has driven the development of modified versions such as PPMOs and Vivo-PMOs to enhance cellular delivery [32]. ASOs are typically distributed through bloodstream or cerebrospinal fluid, depending on the target tissue. Cellular uptake primarily occurs via endocytosis, in which ASOs are internalized into vesicles. To access their RNA targets, they must subsequently escape from endosomes. For targets located in the cytoplasm, endosomal escape is essential before ASOs can bind the RNA. Alternatively, if the target RNA resides in the nucleus, ASO must be translocated into the nucleus either passively through nuclear pores or via active transport mechanisms [36]. However, unmodified nucleotides are susceptible to degradation by nucleases, and their size and negative charge can limit membrane permeability. To address these issues, various chemical modifications have been developed to enhance ASO stability, increase cellular uptake, and improve pharmacokinetics (PK) [37].

Currently, only PMOs have received clinical approval for exon skipping in DMD. PMOs are charge-neutral oligomers characterized by exceptional nuclease resistance and biostability. Their mechanism relies on steric hindrance rather than RNA degradation, as they bind to pre-mRNA at exon–intron junctions or splicing regulatory elements to prevent spliceosome recognition of the targeted exon. Following systemic administration, typically via weekly intravenous (IV) infusions, PMOs enter muscle fibers with limited efficiency, a challenge that remains one of the main therapeutic challenges. Once inside the nucleus, they mask the targeted splicing site, leading to exon exclusion during pre-mRNA processing [22,38]. The resulting transcript lacks the targeted exon and is subsequently translated according to the newly established exon configuration.

Beyond molecular optimization, translating exon-skipping therapies into clinical applications requires robust preclinical validation in relevant animal models. According to international regulatory guidelines (ICH M3(R2), Food and Drug Administration (FDA), and European Medicines Agency (EMA)), preclinical development must include efficacy and safety studies in at least two species (rodent + non-rodent). One rodent and one non-rodent mammal, such as a dog, pig, or non-human primate. This step is essential to assess PK, toxicity, and translational relevance before initiating human clinical trials.

Rodent models, such as the mdx mouse model, are commonly used for proof-of-concept studies in DMD, but they do not fully recapitulate human physiology and disease progression. Larger animal models, including canines and pigs, provide better predictive data for human outcomes. Among these, the recently developed DMD-MMP represents an attractive non-rodent species due to its anatomical, physiological, and metabolic similarities to humans. Its intermediate body size enables repeated sampling, advanced imaging, and chronic dosing studies that are challenging to perform in rodents. Moreover, its longer lifespan and slower growth rate make it particularly suitable for modeling chronic disorders like DMD, which require long-term therapeutic evaluation [27]. Compared to traditional large-animal models such as dogs, DMD-MMPs offer practical advantages: smaller size, easier handling, and lower maintenance costs. DMD-MMPs harboring *DMD* gene mutations have already been generated and display dystrophic features consistent with human pathology. These models hold strong potential for evaluating systemic delivery, biodistribution, and long-term safety of ASO RNA therapies, and cell-based interventions before clinical translation.

In head-to-head terms, DMD-MMPs capture clinically relevant cardiac trajectories and tissue pathology that are challenging to model in mice and often heterogeneous in canine colonies. In the DMD-MMPs, quantitative echocardiography revealed normal LVEF in wild-type animals (~61–62%) and depressed values in age-matched DMD cohorts (mid-40s to low-50s by 12 months), aligning with progressive dysfunction in patients; concomitantly, Masson’s trichrome and H&E staining documented evolving myocardial fibrosis from 6 to 12 months and severe fibrosis by ~30 months [27]. These granular readouts provide a practical framework for prospective assessment of cardiac and diaphragm delivery for ASOs, PPMOs, AOC, and RNA-editing vectors.

Although this model has not yet been used in formal preclinical therapeutic trials, its manageable size and physiological relevance make it an ideal platform for longitudinal studies of oligonucleotide-based therapies. Currently, only an exon 23-deleted DMD-MMP model is available, which limits its use for assessing targeted strategies targeting exons 44 or 51. For example, in the preclinical evaluation of AOC 1044, a PMO conjugated to a transferrin receptor 1 antibody, studies were conducted in patient-derived cells, humanized mouse models, and non-human primates, but not yet in large-animal models [39]. The development of additional DMD-MMP model carrying clinically relevant exon deletions would therefore bridge the translational gap between small-animal data and human trials, supporting the optimization of next-generation oligonucleotide therapies such as AOC and PPMOs [27].

## 3. Evolving Preclinical Animal Models for RNA Therapeutics in DMD

### 3.1. Limitations of Small Animal Models (Mdx Mice)

The mdx mouse has been the most widely used preclinical model for DMD and has provided invaluable insights into dystrophin deficiency and proof-of-concept validation for exon skipping and other RNA-based therapeutics. However, several intrinsic limitations restrict its translational relevance, particularly for advanced RNA therapeutics [40,41].

First, the mdx mouse exhibits a relatively mild disease phenotype compared with human DMD, with near-normal lifespan and efficient muscle regeneration that masks progressive muscle degeneration. Cardiac involvement, a major cause of morbidity and mortality in patients, is modest and late onset in mdx mice, making it difficult to evaluate cardioprotective effects of RNA therapeutics. Second, the small body size and rapid metabolism of mice substantially differ from those of humans, leading to discrepancies in PK, biodistribution, and tissue exposure of ASOs. These differences complicate dose extrapolation and often result in overestimation of therapeutic efficacy.

Moreover, systemic delivery of RNA therapeutics in mdx mice does not adequately reflect clinical challenges such as heterogeneous tissue penetration, long-term toxicity, or immune responses observed in humans. Consequently, while mdx mice remain indispensable for mechanistic studies and early screening, they are insufficient as standalone models for predicting clinical outcomes of RNA therapeutics in DMD.

### 3.2. Large-Animal Models for DMD

To bridge the translational gap between rodent studies and human clinical trials, large-animal models with closer anatomical, physiological, and pathological similarities to humans have been developed. These models are particularly important for evaluating systemic delivery, long-term efficacy, and safety of RNA therapeutics.

#### 3.2.1. Canine Models (GRMD, CXMDJ)

Canine models of DMD, including golden retriever muscular dystrophy (GRMD) and canine X-linked muscular dystrophy in Japan (CXMDJ), closely recapitulate the severe and progressive nature of human DMD. These dogs exhibit early-onset muscle weakness, progressive skeletal muscle degeneration, and clinically relevant cardiomyopathy, making them valuable for translational research [42,43].

Importantly, canine hearts show electrophysiological and structural abnormalities resembling those observed in patients, enabling assessment of cardiac effects of exon-skipping therapies [44,45]. Canine body size also allows for clinically relevant dosing regimens and repeated systemic administration of ASOs, providing more realistic PK and biodistribution profiles than rodent models.

However, practical limitations constrain widespread use of canine models. Long reproductive cycles, variability in disease progression, ethical considerations, and high maintenance costs limit experimental throughput. In addition, genetic and phenotypic heterogeneity among dogs complicates standardized evaluation of therapeutic efficacy. These factors underscore the need for alternative large-animal models that combine translational relevance with experimental feasibility.

#### 3.2.2. Porcine Models and Advantages of MMPs

Porcine models of DMD have long been considered attractive preclinical platforms due to their close anatomical, physiological, and metabolic similarities to humans. To enhance translational relevance beyond rodent models, genetically engineered DMD pigs have previously been generated in domestic pigs and minipigs using targeted gene disruption approaches [27,46].

However, a critical limitation of these early porcine DMD models has been their extremely short lifespan. Most of these models were produced using somatic cell nuclear transfer (SCNT)-based gene modification techniques, which are known to be associated with epigenetic abnormalities. Such epigenetic dysregulation is thought to contribute to severe developmental defects and early lethality. Although subsequent methodological refinements have partially ameliorated these issues and extended survival, the reported lifespan of these DMD pigs has remained limited, with a maximum survival of approximately 3–4 months of age [47].

This lifespan corresponds to the human pre-adolescent stage and is insufficient to model the progressive nature of DMD [48]. In particular, it precludes meaningful investigation of secondary cardiomyopathy, which develops over time and has become a major determinant of prognosis in DMD patients. Consequently, despite their anatomical advantages, earlier porcine DMD models have not been suitable for long-term pathophysiological studies or therapeutic development targeting cardiac involvement.

In contrast, the MMPs, represents a significant advancement in porcine DMD modeling [27]. Using CRISPR/Cas9-mediated genome editing, a mutation was introduced into exon 23 of the *DMD* gene, followed by establishment of a stable breeding colony through natural mating. This strategy avoided the reliance on SCNT and enabled the generation of a genetically and epigenetically stable DMD pig line.

As a result, DMD-MMP model exhibit markedly prolonged survival, with confirmed lifespans extending up to 2.5 years. Considering that MMPs reach sexual maturity at approximately 5–8 months of age and attain adult body size by around 12 months, this longevity allows modeling of disease stages corresponding to adolescence and adulthood in humans. This temporal window is particularly critical for evaluating disease progression, including the development of cardiomyopathy, as well as for assessing long-term efficacy and safety of RNA therapeutics.

Collectively, the DMD-MMP model overcomes key limitations of earlier porcine models and provides a physiologically and temporally relevant large animal platform for translational research, particularly for RNA-based therapeutic strategies targeting both skeletal and cardiac muscle.

### 3.3. Translational Relevance of MMPs for RNA Therapeutics

MMPs represent an emerging and highly relevant platform for translational studies of RNA therapeutics in DMD. Their body size enables clinically relevant dosing volumes and administration routes, allowing accurate evaluation of PK and pharmacodynamic relationships that are difficult to extrapolate from rodent models [26].

Biodistribution studies in pigs provide critical information on tissue-specific uptake of ASOs, particularly in skeletal muscle and the heart, where therapeutic delivery remains challenging. The prolonged lifespan and slower metabolic rates of MMPs facilitate long-term assessment of drug accumulation, durability of exon skipping effects, and potential off-target toxicity.

Importantly, the porcine cardiovascular system closely resembles that of humans in terms of anatomy, conduction properties, and susceptibility to cardiomyopathy [49,50]. This feature enables comprehensive evaluation of cardiac involvement in DMD and the effects of RNA therapeutics on cardiac structure and function. As cardiomyopathy has become a leading cause of death in DMD patients due to advances in respiratory management, the ability to assess cardiac outcomes in a translationally relevant model is of increasing importance.

Although canine models develop clinically relevant cardiomyopathy, variability in onset and progression can complicate quantitative longitudinal assessment, DMD-MMPs have been shown to exhibit a clear and progressive decline in cardiac function within a clinically relevant time frame [27,44,45]. A significant reduction in LVEF has been reported in DMD-MMPs compared with wild-type animals at 6 and 12 months of age, indicating progressive cardiac impairment. Notably, 12 months of age in MMPs roughly correspond to early adulthood in human DMD patients, at which time LVEF in humans is estimated to decline to around 40%. The magnitude and timing of LVEF reduction observed in DMD-MMPs closely mirror the clinical course of cardiomyopathy in human DMD, underscoring the translational relevance of this model.

Together, these findings suggest that MMPs enable earlier and more quantitative assessment of DMD-associated cardiomyopathy than canine models, while maintaining physiological and anatomical similarity to the human cardiovascular system, thereby providing a practical and highly relevant platform for evaluating cardiac outcomes and therapeutic interventions.

Collectively, MMPs fill a critical gap between rodent and clinical studies and offer a promising preclinical platform for advancing RNA therapeutics toward successful clinical translation in DMD.

## 4. Clinical Advances and Successes

The development of PMO-based ASOs has led to several exon-skipping therapies reaching clinical use for DMD. These therapies target the most frequently mutated exons in the *DMD* gene, such as exons 51, 53, and 45, which together account for a significant proportion of patients. Eteplirsen (Exondys 51) was the first PMO approved by the U.S. FDA in 2016 for patients amenable to exon 51 skipping, representing approximately 13% of the DMD population [51]. Subsequent approvals include Golodirsen (Vyondys 53) and Viltolarsen for exon 53 skipping, and Casimersen (Amondys 45) for exon 45 skipping, thereby expanding the proportion of patients eligible for treatment [52,53,54]. Despite these milestones, the clinical benefits remain modest, as these drugs generally produce low levels of dystrophin restoration (1–10% of normal) and do not halt disease progression [38,52].

### 4.1. Eteplirsen

Eteplirsen (Exondys 51; Sarepta Therapeutics, Cambridge, MA, USA) was the first exon-skipping ASO drug approved by the FDA in 2016. It is a PMO-based drug developed to target exon 51 in DMD patients. Eteplirsen was tested in a smaller group of patients, ultimately evaluating doses of 30 mg/kg and 50 mg/kg in 12 patients in an open-label study, and the FDA granted Eteplirsen accelerated approval based on dystrophin restoration data [22]. Biopsies from Eteplirsen-treated patients showed clear levels of exon skipping and increased dystrophin production. After 48 weeks of weekly IV administration, patients exhibited increased dystrophin-positive fibers. Extended treatment over 96 weeks further improved efficacy, showing an average of 1.091% exon skipping and 0.63% dystrophin production, with 27.38% dystrophin-positive fibers [38]. These molecular improvements translated into functional benefits, as long-term treatment reduced the annual decline in ambulatory capacity and pulmonary function compared to natural history controls. Real-world data from patients treated for 4–7 years showed a 2.09-year delay in loss of ambulation, a 5.72-year delay before requiring continuous ventilation, and improved overall survival by more than 5 years. Eteplirsen has generally exhibited a favorable safety profile, with adverse events primarily related to infusion procedures, including catheter-related complications (8.1%) and infusion reactions (8.9%), as reported in the EVOLVE study (NCT06606340). Despite these outcomes, efficacy remains limited, with low dystrophin restoration and modest clinical benefit. This limitation, alongside the lack of robust placebo-controlled evidence, led the EMA to reject Eteplirsen’s approval in 2018. To address these challenges, the ongoing MIS51ON trial (NCT03992430) is evaluating higher doses (100–200 mg/kg) to optimize therapeutic response. The EVOLVE study is a multicenter clinical trial designed to assess the safety, PK, and dystrophin restoration of next-generation exon-skipping therapies in patients with DMD [29,38].

### 4.2. Golodirsen

Golodirsen (Sarepta Therapeutics) is a PMO designed to induce skipping of exon 53 in the *DMD* gene, and theoretically, 7.7% of DMD patients would benefit from Golodirsen [28]. The drug received conditional approval from the FDA in 2019, supported by results from a phase 1/2 clinical trial (NCT02310906), which reported 18.95% exon 53 skipping, 1.02% dystrophin protein restoration, and 10.47% dystrophin-positive fibers after 48 weeks of therapy. Long-term data from patients treated for 3 years indicate a slower decline in motor and respiratory function compared with natural history controls, with an estimated 2.4-year delay in the loss of ambulation and approximately a 50% reduction in the annual rate of pulmonary decline. Golodirsen has demonstrated a favorable safety profile. Although adverse events were reported, no clear causal relationship with the study drug was established. Its long-term efficacy and safety continue to be evaluated in ongoing trials, including the phase 3 ESSENCE study (NCT02500381) and the phase 4 EVOLVE study (NCT06606340). The ESSENCE study is a randomized, double-blind, placebo-controlled Phase III trial evaluating the efficacy and safety of exon 45 and exon 53 skipping PMOs in patients with DMD amenable to these exon-skipping strategies [34,38].

### 4.3. Viltolarsen

Viltolarsen, (NS Pharma, Paramus, NJ, USA), is a PMO targeting exon 53 of the *DMD* gene. The drug has been approved in Japan and by the FDA based on evidence from phase 2 clinical trials (NCT02740972) and their open-label extension (NCT03167255). In these studies, weekly IV administration at 80 mg/kg for 25 weeks led to an average dystrophin expression of 5.9%, accompanied by functional improvements compared to baseline and external matched controls over a four-year observation period [53]. However, these promising early results were not fully replicated in the phase 3 RACER53 trial (NCT04060199) involving 77 patients, in which only a slight improvement in the primary functional endpoint was observed, without reaching statistical significance versus placebo. Viltolarsen was generally well tolerated, with adverse events reported, but investigators did not attribute these events to the study drug. The most frequent being cough and nasopharyngitis. The long-term therapeutic potential of viltolarsen continues to be assessed in the open-label RACER53-X extension study (NCT04768062) [38,55].

### 4.4. Casimersen

Casimersen (Amondys 45, Sarepta Therapeutics) is a PMO exon-skipping therapy targeting exon 45 of the *DMD* gene. The drug received FDA conditional approval in 2021, following evidence from a randomized, double-blind, placebo-controlled phase 1/2 trial (NCT02530905) [54]. After 48 weeks of treatment, patients demonstrated significant molecular improvements, including increases in exon 45 skipping from 0.41% to 2.02%, dystrophin expression from 0.93% to 1.74%, and dystrophin-positive fibers from 6.46% to 15.26%. Casimersen exhibited a favorable safety profile, with no major treatment-related adverse events reported. Ongoing investigations, such as the phase 4 EVOLVE trial (NCT06606340), aim to assess its long-term safety and clinical efficacy further. Additionally, Casimersen has shown potential benefits in patients with frame-shifting duplications amenable to exon 45 skipping, expanding its therapeutic applicability [34,38].

### 4.5. Brogidirsen

A phase 1/2 clinical trial evaluated Brogidirsen, a dual-targeting ASOs designed for exon 44 skipping, in patients with DMD. This study aimed to assess safety, PK, exon-skipping efficacy, and dystrophin restoration, and to identify potential biomarkers and explore functional outcomes. Brogidirsen is a novel ASO composed of two directly linked 12-mer PMOs, providing enhanced affinity and stability. PMOs are resistant to nuclease degradation, bind strongly to pre-mRNA, and preferentially target regenerating myofibers. The first trial enrolled six ambulant male patients aged 4–13 years in Japan between December 2019 and October 2021. Brogidirsen was found to be safe and well-tolerated up to 80 mg/kg for 24 weeks, with no serious adverse events or study discontinuations. Dystrophin levels increased in a dose-dependent manner, reaching 16.63% of normal at 40 mg/kg and 24.47% at 80 mg/kg after 24 weeks, exceeding levels reported for previous exon-skipping therapies. Exon 44-skipping efficiency also improved in all biopsied muscles. Although no clear improvement in motor function was observed, patients maintained or slightly improved performance, suggesting functional stabilization. Reductions in potential biomarkers associated with muscle necrosis, PADI2, titin, and myomesin 2 were observed, indicating improved muscle pathology. In vitro, brogidirsen demonstrated robust exon-skipping activity in patient-derived urine cells reprogrammed toward a myogenic lineage through MYOD1 overexpression. These cells provide a noninvasive, patient-specific model to evaluate dystrophin restoration and RNA-based therapeutic efficacy. Overall, this first-in-human trial showed a favorable safety profile and promising molecular efficacy, supporting further development of brogidirsen as an exon 44-skipping therapy for DMD [24]. An overview of the main ASOs chemistry and conjugation strategies currently explored for DMD is provided in Table 1.

### 4.6. Next-Generation Delivery Strategies for Exon-Skipping ASO

The current generation of PMO-based ASOs, although approved for DMD, shows limited efficacy in exon skipping and dystrophin restoration. Consequently, research efforts have focused on developing next-generation ASOs with improved chemical properties and molecular conjugations to enhance therapeutic performance. The first involves optimizing dosing regimens, with higher doses of first-generation PMO compounds being tested to improve therapeutic outcomes. The second strategy centers on developing second-generation PMOs that incorporate chemical modifications and bioconjugation techniques. Chemical modifications aim to enhance stability and binding affinity, while bioconjugation to peptides, antibodies, or other molecular carriers seeks to improve cellular uptake, facilitate endosomal escape, and promote nuclear import to the splicing site [29]. Several of these next-generation ASOs are currently under clinical investigation for DMD (Table 1). Preliminary data indicate improved molecular efficacy compared to first-generation PMOs. However, their safety profiles require careful evaluation. These advances highlight both the progress made and the challenges that remain in the development of more effective exon-skipping therapies for DMD and related NMD [38,56].

CPPs represent a promising strategy to overcome one of the major limitations of exon-skipping therapy in DMD: the poor uptake of PMOs in muscle tissue, particularly the heart. CPPs are short amino acid sequences, typically rich in positively charged residues such as arginine and lysine, that enable efficient intracellular delivery of conjugated molecules. When conjugated to PMOs to form PPMOs, these peptides significantly enhance cellular uptake and nuclear delivery, leading to improved exon skipping and dystrophin restoration at lower doses than unconjugated PMOs [57]. CPPs promote cellular uptake primarily through endocytic pathways. Furthermore, cyclic CPPs exhibit superior stability against proteolytic degradation compared to linear peptides and can facilitate cytoplasmic delivery by promoting endosomal escape. Several CPP-based approaches are currently under clinical evaluation. SRP-5051 (Vesleteplirsen), developed by Sarepta Therapeutics, demonstrated higher dystrophin production compared with Eteplirsen in early clinical studies [34,38]. Despite these efficacy results, hypomagnesemia was observed in nearly all 62 of the treated patients, with 4 cases classified as serious. Some cases of hypomagnesemia persisted even after treatment discontinuation and were associated with reduced glomerular filtration rate, indicating renal toxicity. Additionally, hypokalemia was reported in almost half of the patients treated. Given these concerning safety findings, Sarepta Therapeutics announced the discontinuation of the SRP-5051 program in November 2024. Similarly, PGN-EDO51 from PepGen showed a 4-fold increase in exon-skipping capacity in preclinical models, but clinical evaluation also reported cases of hypomagnesemia. To mitigate these safety concerns, newer platforms such as Endosomal Escape Vehicles (EEVs) have been developed. EEVs, composed of cyclic arginine-rich CPPs, promote both cellular internalization and efficient endosomal escape. ENTR-601-44, an EEV-conjugated PMO targeting exon 44, has shown enhanced exon skipping in skeletal and cardiac muscle in both mouse models and non-human primates. Collectively, CPP-based delivery systems hold great potential to improve the efficacy of exon-skipping therapies, though optimization is required to address associated toxicity risks [34,38].

Despite recent advances, several critical challenges continue to limit the long-term efficacy and accessibility of exon-skipping therapies. One major limitation is the poor systemic delivery of ASOs, particularly PMOs, to cardiac tissue [24,28,58]. This represents a significant concern, as cardiomyopathy is a leading cause of mortality in DMD. The limited efficiency is largely due to the physicochemical properties of PMOs, which hinder their uptake by cardiomyocytes and result in subtherapeutic concentrations in the heart. To overcome this barrier, next-generation exon-skipping ASO therapeutics are in development with new chemistries and bioconjugations, such as PPMOs, CPPs, AOCs, ligand-targeted oligonucleotides, and dual-targeting ASOs, which are currently under investigation [38,39]. Nevertheless, currently approved ASO therapies generally restore only low levels of dystrophin in patient biopsies, reflecting persistent challenges in delivery, tissue penetration, and splice-switching efficiency [20].

However, a major challenge remains unresolved: the cost and overall therapeutic potential. Reported dystrophin restoration levels in clinical studies generally fall within the low single-digit percentage range of normal dystrophin expression, although values vary substantially depending on the targeted exon, treatment duration, muscle analyzed, and quantification methodology. To contextualize this variability, Table 2 summarizes representative exon-skipping ASO applications across different mutation classes, model systems, and assessment methods. Efficacy varies substantially depending on the targeted exon, the underlying mutation, the muscle group analyzed, and the methodology used for dystrophin quantification, including Western blotting, immunofluorescence, or mass spectrometry. In addition, inter-patient variability related to disease stage, age at treatment initiation, and baseline muscle pathology further contributes to heterogeneous molecular and functional responses to PMO-based therapies. These therapies prolong the time to respiratory and ambulatory support by only a few years. Moreover, given the modest efficacy achieved to date, the treatment cost is prohibitively high, raising important concerns regarding cost-effectiveness and healthcare system sustainability [18,20,38].

As illustrated in Table 2, exon-skipping efficacy varies according to genotype context, model organism, and experimental methodology. Protein-level measurements, including WB, IF, and Mass spectrometry, differ in sensitivity and quantitative accuracy, while RNA-based assays primarily reflect exon-skipping efficiency rather than functional protein recovery. These methodological differences, together with biological variability across tissues and patient cohorts, contribute to the wide range of dystrophin restoration values reported in the literature and underscore the need for cautious interpretation of efficacy metrics across studies [20,59,60].

## 5. Discussion

### 5.1. Translational Value of the DMD-MMP Model

The successful clinical translation of RNA-based therapeutics for DMD depends not only on advances in molecular design but also on the availability of predictive preclinical models. While rodent models remain essential for mechanistic studies and early therapeutic screening, their limited body size and physiological differences restrict their predictive value for systemic delivery, tissue distribution, and long-term safety assessment of RNA therapeutics. These limitations have increased interest in large-animal models that better recapitulate human anatomy and disease progression. Among these, the MMP, the smallest miniature pig strain with an adult body weight of approximately 20 kg, provides practical and translational advantages. Its size facilitates handling and experimental procedures while reducing the dose requirements for systemically delivered therapeutics. In addition, the physiological similarities between pigs and humans support clinically relevant evaluation of therapeutic efficacy and biodistribution. Nevertheless, MMP models also present limitations, including species-specific differences in lifespan, growth kinetics, and disease progression, as well as logistical and ethical constraints associated with large-animal studies. Despite these challenges, the DMD-MMP model enables clinically relevant administration routes, longitudinal functional assessment, and repeated systemic dosing, making it a valuable translational platform for evaluating the efficacy and safety of emerging RNA-based therapeutic strategies.

From a translational perspective, the genome-edited DMD-MMP model helps address this long-standing delivery challenge by enabling repeated sampling, cross-sectional imaging, and serial biopsies under clinically relevant dosing paradigms. This platform therefore provides a valuable system for evaluating cardiac exposure and long-term tolerability of next-generation ASO chemistries and RNA-editing platforms, particularly for therapies targeting the heart and diaphragm [27].

### 5.2. Limitations of ASO-Mediated Exon-Skipping Therapies

Inter-individual variability represents a major challenge for the clinical implementation of RNA-based therapies in DMD. Therapeutic responses are influenced by several factors, including the specific mutation in the *DMD* gene, differences in exon-skipping efficiency, the level of restored dystrophin expression, disease stage at treatment initiation, and baseline muscle pathology. These sources of heterogeneity complicate the evaluation of clinical efficacy and limit the applicability of uniform treatment paradigms. Consequently, personalized therapeutic strategies are increasingly required. Precision medicine approaches, including genotype-driven therapy selection, early intervention in pre-symptomatic or minimally affected patients, and the use of molecular and imaging biomarkers to stratify patients and monitor treatment response, are likely to play an important role in optimizing treatment outcomes. In addition, adaptive or small-cohort clinical trial designs may be necessary to adequately evaluate mutation-specific RNA therapies and capture clinically meaningful benefits in genetically defined subpopulations [61,62,63].

In addition, exon-skipping therapies require lifelong administration. Because ASOs do not integrate into the genome and have relatively short half-lives, repeated IV infusions are necessary to maintain therapeutic levels [37]. In clinical practice, patients often undergo weekly or biweekly IV administrations, which impose a substantial treatment burden on patients and caregivers and may negatively affect quality of life and treatment adherence, particularly in pediatric populations.

Nevertheless, ASO-mediated exon skipping remains the most mature and clinically validated gene-modulating strategy for DMD to date and provides a strong foundation for next-generation approaches, including multi-exon skipping and improved delivery systems.

### 5.3. Emerging Strategy in RNA-Based Therapies on RNA Editing for NMD

RNA editing using ADARs has emerged as a promising therapeutic strategy for correcting point mutations at the RNA level, offering a precise and transient alternative to permanent genome editing. In ADAR-mediated RNA editing, the guide RNA (gRNA) hybridizes to the target mRNA near the edited adenosine, creating a double strand RNA substrate. ADARs catalyze the deamination of adenosine (A) to inosine (I), which is interpreted as guanosine (G) during translation. This reaction enables site-specific A → G substitutions on target mRNA [64,65].

**Figure 3:**
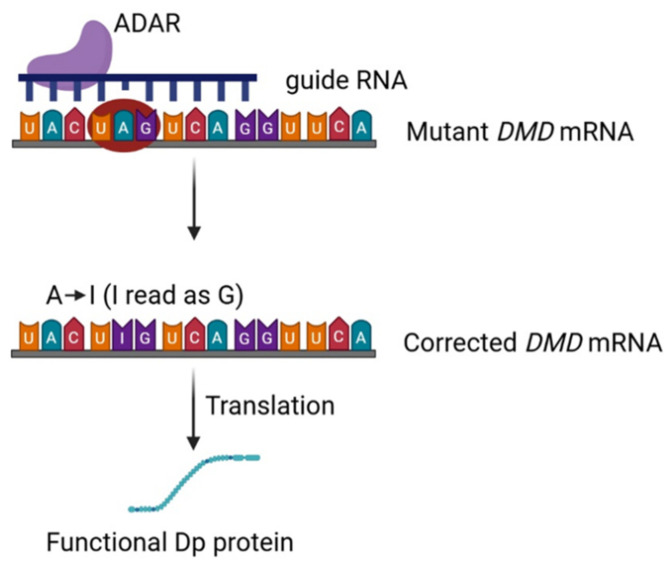
Principle of ADAR based RNA-editing strategies applied to DMD. RNA editing enables correction of pathogenic point mutations at the transcript level without altering genomic DNA revealing a premature UAG stop codon. gRNAs bind to mRNA and a mismatch appears between point mutation and the gRNA. This gRNA recruit RNA-editing enzymes, such as ADAR, to specific adenosine residues within mutant DMD transcripts, inducing targeted base conversion (e.g., A-to-I editing). This modification can restore a functional codon, allowing translation of a corrected dystrophin protein. RNA editing acts transiently at the RNA level and requires sustained or repeated delivery to maintain therapeutic effect. Red circle represents a premature stop codon. ADAR (adenosine deaminase acting on RNA); *DMD* (*DMD* gene).

This approach is particularly relevant to the 10–15% of DMD patients who carry nonsense mutations introducing premature stop codons such as UGA, UAA, or UAG, which lead to truncated and non-functional dystrophin proteins [66]. In specific cases, particularly when a UAG codon contains an editable adenosine, ADAR-mediated A → I editing can convert a stop codon into a coding codon (e.g., UAG → UGG, tryptophan), potentially restoring translation of full-length or partially functional dystrophin. However, because ADAR enzymes catalyze only A → I conversions, RNA editing can correct only a subset of nonsense mutations (e.g., UAG → UGG), whereas codons such as UAA or UGA are poorly editable or not editable [67]. The principle of RNA-editing strategies applied to DMD is schematically depicted in Figure 3. Therefore, RNA editing is applicable only to a subset of nonsense mutations, making it a strategy that requires careful selection of suitable cases based on codon constraints rather than a broadly generalizable approach.

The ADAR family comprises several RNA-editing enzymes, among which ADAR1 and ADAR2 are catalytically active. Among these, ADAR1 and ADAR2 are catalytically active and naturally expressed in various tissues, including the brain and muscle [65].

Therapeutic strategies leverage this endogenous machinery by directing ADAR enzymes to specific RNA targets using engineered gRNA or by expressing ADAR-fusion proteins such as bacteriophage coat protein (MCP)-ADAR, Cas13-ADAR that enhance both specificity and editing efficiency. These systems enable precise correction of single-point mutations without modifying the genome, thereby minimizing the risk of off-target DNA alterations [68].

In most studies, RNA-editing systems are delivered using AAV vectors to enable sustained expression in post-mitotic tissues such as skeletal and cardiac muscle, although alternative delivery platforms remain under investigation [68,69,70]. However, AAV-mediated delivery raises important immunogenicity considerations. Pre-existing neutralizing antibodies against AAV capsids may limit transduction efficiency and restrict the possibility of redosing. In addition, sustained expression of exogenous or engineered RNA-editing enzymes may trigger adaptive immune responses [20,71].

Compared with CRISPR-Cas-based genome editing, ADAR-mediated RNA editing does not introduce double-strand DNA breaks and does not permanently modify the genome. This transient and reversible mode of action may reduce the risk of irreversible off-target DNA mutations. However, it also necessitates sustained or repeated delivery to maintain therapeutic benefit, thereby shifting the risk profile from genomic permanence to vector durability and immune tolerance [23,65,69].

Proof-of-concept studies have demonstrated successful A→I editing of UAG stop codons into UGG in vitro and in vivo, including in the mdx mouse model of DMD [64]. More recently, optimized U7 small nuclear RNA (snRNA) scaffolds with strong synthetic promoters have further enhanced ADAR-mediated editing efficiency. In a Hurler syndrome mouse model, this approach enabled robust RNA editing in the brain after a single systemic AAV injection, outperforming circular gRNA systems [72]. U7 snRNA serves as a scaffold for gRNA delivery, enhancing the precision and efficiency of ADAR recruitment. Such strategies hold strong potential for adaptation to DMD therapies, particularly for nonsense mutations with favorable codon contexts for A→I editing.

### 5.4. Comparison of Oligonucleotide Therapeutics and RNA-Based Therapies

RNA-based therapeutics, including exon-skipping ASO and RNA editing approaches, offer a distinct therapeutic paradigm compared with DNA-editing or protein-replacement strategies, owing to their sequence specificity, reversibility, and lack of permanent genomic modification [65,70]. These modalities enable mutation-tailored interventions at the transcript level. In DMD, exon-skipping ASO restores the open reading frame in defined mutation classes by targeting specific exons, whereas RNA editing can, in principle, correct diverse single-nucleotide variants, including nonsense mutations, thereby enabling restoration of full-length dystrophin without altering the genomic DNA. However, ASO-mediated exon skipping is inherently mutation-class specific, with patient coverage partitioned across individual exon targets, requiring multiple drugs to address the broader DMD population. Despite this limitation, exon skipping remains the most clinically advanced RNA therapeutic approach in DMD, supported by regulatory approvals of PMOs and extensive clinical experience. Delivery constraints, particularly to cardiac and respiratory muscles, have driven the development of conjugated platforms such as PPMOs and AOCs.

RNA editing offers broader theoretical mutation coverage by enabling single-nucleotide correction at the RNA level, potentially applicable across heterogeneous *DMD* genotypes. Preclinical studies using Cas13- or ADAR-based editors have demonstrated efficient transcript correction and dystrophin rescue in humanized DMD mouse models, including in skeletal and cardiac muscle. Despite its conceptual appeal, RNA editing is associated with several technical and biological limitations that must be carefully considered. Off-target RNA editing remains a concern due to unintended nucleotide conversions in partially complementary transcripts. In addition, the transient nature of RNA editing necessitates sustained editor expression or repeated delivery to maintain therapeutic benefit. Both RNA editors and their delivery vectors, most commonly AAV, may trigger immune responses that limit transduction efficiency and redosing. These liabilities necessitate stringent control of AAV specificity, expression levels, and delivery modalities in any translational RNA-editing strategy [20,69].

In contrast to CRISPR-based genome editing and AAV-mediated gene replacement, RNA editing offers a fundamentally distinct risk-benefit profile. Genome editing enables permanent correction of pathogenic mutations but carries the risk of irreversible off-target DNA modifications and long-term genomic instability [23,71]. AAV gene therapy, while clinically advanced, is constrained by vector packaging limits, dose-dependent toxicity, and sustained transgene overexpression. However, this reversibility comes at the cost of durability, as therapeutic benefit depends on continued editor expression or repeated administration. Quantitatively, current RNA-editing approaches generally achieve partial correction efficiencies at the transcript level, which may be sufficient for functional rescue in certain contexts but remain lower than the dystrophin levels typically reported with gene replacement strategies. As such, RNA editing should be viewed not as a direct replacement for DNA-based therapies, but as a complementary strategy, particularly suited for applications where safety, reversibility, and precise mutation targeting are prioritized.

As summarized in Table 3, exon-skipping ASO therapies and RNA-editing strategies exhibit complementary therapeutic profiles rather than competing paradigms. Exon skipping currently represents the most clinically mature RNA-based therapeutic strategy for DMD, supported by multiple regulatory approvals and accumulated safety data, but remains limited by exon-specific applicability, modest dystrophin restoration in human biopsies, and suboptimal delivery to cardiac and diaphragmatic muscle. In contrast, RNA editing offers broader mutational flexibility and the conceptual potential to restore near-native dystrophin at the transcript level; however, it remains preclinical in DMD and is constrained by delivery efficiency, durability, and safety considerations. Importantly, these approaches address distinct therapeutic needs and may ultimately be deployed in a genotype and disease-stage-dependent manner. Together, these observations support a therapeutic landscape in which exon skipping serves as a near-term disease-modifying strategy, while RNA editing may emerge as a targeted option for selected genotypes as delivery technologies, editor specificity, and regulatory pathways continue to mature [20,69].

### 5.5. Overall Challenges and Future Prospects of RNA-Based Therapeutics

Because ASO therapies do not integrate into the genome, sustained benefit requires repeated administration, and the long-term consequences of chronic exposure remain incompletely characterized [20]. Collectively, these observations underscore the need for extended post-marketing surveillance, improved delivery strategies that reduce dosing frequency, and the development of predictive biomarkers to monitor cumulative toxicity over time.

Clinical experience with first-generation PMO-based exon-skipping therapies has generally demonstrated a favorable safety profile under long-term administration, with most adverse events related to infusion procedures rather than direct drug toxicity. Reported adverse events have mainly included infusion-related reactions and catheter complications, without consistent evidence of severe organ toxicity [38,73]. In contrast, next-generation conjugated ASOs, particularly PPMOs, have revealed dose-dependent safety concerns in clinical development, including hypomagnesemia, electrolyte disturbances, and reductions in estimated glomerular filtration rate, suggesting potential renal tubular toxicity [29,74]. While these findings remain under investigation, they indicate that enhanced cellular uptake strategies may introduce new class-specific safety liabilities. Immune responses against AAV vectors or engineered delivery systems may also limit redosing and long-term durability. Continued pharmacovigilance and careful dose optimization will therefore be essential to distinguish manageable safety signals from platform-limiting toxicities.

Efficient and durable delivery to cardiac and diaphragmatic muscle remains a central translational challenge and continues to limit the therapeutic index of current RNA-based platforms. RNA editing, while still in its preclinical stages for DMD, holds great promise for NMD, particularly in cases involving nonsense mutations. Ongoing efforts focus on improving delivery systems, increasing editing efficiency, and minimizing off-target events [29]. As the field matures, RNA editing could become a valuable complement to exon-skipping and other RNA-based therapies within a combinatorial therapeutic framework [69].

Future research efforts in RNA-based therapies for DMD should prioritize several key directions to overcome current limitations. First, the development of next-generation delivery platforms capable of achieving efficient and sustained targeting of cardiac and diaphragmatic muscle remains a critical unmet need. Second, strategies aimed at increasing intracellular stability and potency, thereby reducing dosing frequency, are essential to improve long-term tolerability and patient adherence. Third, systematic evaluation of long-term safety, including cumulative toxicity and immunological effects, will be required to support lifelong treatment paradigms. Finally, the integration of predictive biomarkers and patient stratification frameworks will be necessary to align therapeutic approaches with individual disease trajectories and maximize clinical benefit. Together, these priorities will be central to the successful translation of RNA-based strategies into durable and broadly applicable treatments for DMD.

Taken together, these advantages and challenges highlight both the transformative potential and the complexity of RNA-based strategies in DMD. Addressing these challenges will require not only advances in molecular design and delivery technologies, but also the appropriate selection of preclinical models that enable longitudinal evaluation of efficacy, biodistribution, and cumulative toxicity under clinically relevant dosing regimens. Ongoing innovation in delivery technologies, safety profiling, and personalized design will be critical to realizing their full therapeutic potential.

## Figures and Tables

**Table 1:** ASOs targeting exons for DMD: approved therapies and ongoing clinical trial. The table summarizes exon-specific splice-switching ASOs targeting DMD pre-mRNA, including their chemical backbone, delivery strategies, sponsors, and clinical development status. ASO (antisense oligonucleotide); PMO (phosphorodiamidate morpholino oligomer); PPMO (peptide-conjugated PMO); CPP (cell-penetrating peptide); Fab (antibody fragment antigen-binding domain). Conjugation strategies are designed to enhance muscle uptake and intracellular delivery, particularly to cardiac and diaphragmatic muscle.

ASO Name	Mechanism	Chemical	Sponsor	Clinical Trials	Status
Eteplirsen	Exon 51	PMO	Sarepta Therapeutics	………………………	Approved by FDA in 2016
Golodirsen	Exon 53	PMO	Sarepta Therapeutics	………………………	Approved by FDA in 2019
Viltolarsen	Exon 53	PMO	NS Pharma/Nippon Shinyaku	………………………	Approved by FDA + Japan in 2020
Casimersen	Exon 45	PMO	Sarepta Therapeutics	………………………	Approved by FDA in 2021
SRP-5051	Exon 51	PPMO, CPP	Sarepta Therapeutics	NCT04004065	Phase II terminated (2025)
WVE-N531	Exon 53	Stereopure ASO	Wave Life Sciences	NCT04906460	Phase I/II (2025)
PGN-EDO51	Exon 51	Enhanced delivery oligo, CPP	PepGen	NCT06833931	Phase II (2025)
Dyne-251	Exon 51	PMO + ligand (Fab) conjugate	Dyne Therapeutics	NCT05524883	Phase I/II (2025)
Brogidirsen	Exon 44	PMOs, dual targeting	Nippon Shinyaku	NCT05135663	Phase II (2025)

**Table 2:** Variability of exon-skipping ASO drug efficacy in DMD: mutation context, experimental models, and assessment methodologies. ASO (antisense oligonucleotide); DMD (Duchenne muscular dystrophy); PMO (phosphorodiamidate morpholino oligomer).

Model Organism	Genotype/Mutation Context	PMO (Drug/Target Exon)	Delivery Route	Efficacy Assessment Method	Reported Dystrophin Level
Human	Out-of-frame deletion amenable to exon 51 skipping	Eteplirsen (Exon 51)	Weekly intravenous (IV)	Western blot (WB)/Immunofluorescence (IF)	Low single-digit %
Human	Deletion amenable to exon 53 skipping	Golodiresen/Viltolarsen (Exon 53)	Weekly IV	WB/IF/Mass spectrometry	Low single-digit %
Human	Duplication or deletion amenable to exon 45 skipping	Casimersen (Exon 45)	Weekly IV	WB/IF	Low single-digit %
Human (Phase 1/2)	Exon 44 amenable deletion	Brogidirsen (Exon 44)	IV	WB/IF	Up to ~20% (small cohort)
Mdx mouse	Nonsense mutation in exon 23	PMO (Exon 23)	Systemic injection	WB/IF	Variable; higher than human

**Table 3:** Comparative analysis: ASO-mediated exon skipping vs. RNA editing in DMD. ASO (antisense oligonucleotide); DMD (Duchenne muscular dystrophy); PMO (phosphorodiamidate morpholino oligomer); FDA (Food and Drug Administration); IV (intravenous); PPMO (peptide-conjugated phosphorodiamidate morpholino oligomer); AAV (adeno-associated viruses); AOC (antibody–oligonucleotide conjugate; EEV(Endosomal Escape Vehicle); CMC (Chemistry, Manufacturing and Controls); IND (Investigational New Drug).

	ASO Exon Skipping	RNA Editing
Therapeutic goal	Mask splice motifs to skip a selected exon and restore the reading frame. Objectives are truncated but partially functional Dp.	Base-level correction of RNA to restore native reading frame/protein without changing DNA.
Maturity/clinical status	Most clinically advanced RNA therapy in DMD. Multiple exon-specific PMO drugs have FDA accelerated approvals with ongoing Phase 3/4 data.	Preclinical for DMD; early clinical-stage RNA-editing programs reported in non-DMD indications. Robust in vivo efficacy demonstrated in DMD mouse models (e.g., Cas13-based RNA editing).
Applicability to genotypes	Exon-specific. Covers defined deletion/duplication patterns. Multiple drugs needed to cover the population.	Potentially broad, can address nonsense and select missense/frameshift mutations at the RNA level with tailored guides/editors.
Molecular outcome	Produces internally truncated Dp. Typically, low % protein restoration in human biopsies.	Aims to restore near-native dystrophin following transcript correction, high on-target editing reported in mouse skeletal and cardiac muscle.
Dosing/durability	Chronic IV dosing (weekly). Tissue uptake, especially cardiac, is limited. Delivery enhancers (PPMO) improve uptake but introduce safety trade-offs like hypomagnesemia with some PPMO.	Editing persists only while the editor is expressed; durability depends on delivery modality (AAV or Lipid nanoparticle) and expression kinetics. Redosing strategies are under active optimization.
Safety considerations	PMO: favorable history, PPMOs increase efficacy but have magnesium issues under investigation.	Risk of off-target RNA edits, innate immune sensing of guides/editors, and vector-related risks. Nonetheless, no DNA change, which may reduce long-term genomic risk.
Cardiac/diaphragm delivery	Historically suboptimal. Newer AOC/EEV platforms show stronger cardiac/diaphragm delivery in preclinical and early clinical readouts.	In mice, systemic Cas13 RNA editing achieved high editing in heart and diaphragm.
Manufacturing/CMC	Established PMO manufacturing.	Complex (editor enzyme + guide. Often vectorized). Multiple components increase CMC burden and regulatory scrutiny.
Regulatory trajectory	Multiple accelerated approvals, confirmatory evidence ongoing.	First RNA-editing INDs have been cleared in non-DMD indications; DMD-directed programs remain preclinical.

## Data Availability

No new data were created or analyzed in this study. Data sharing is not applicable to this article.

## Abbreviations

DMD	Duchenne muscular dystrophy
NMD	neuromuscular disease
ASO	antisense oligonucleotide
ADAR	adenosine deaminase acting on RNA
PMO	phosphorodiamidate morpholino oligomer
PPMO	peptide-conjugated phosphorodiamidate morpholino oligomer
AOC	antibody–oligonucleotide conjugate
MMPs	microminipigs
CK	creatine kinase
LVEF	left-ventricular ejection fraction
DMD-MMPs	Duchenne muscular dystrophy model of microminipigs
ROS	reactive oxygen species
AAV	adeno-associated viruses
IV	intravenous
WB	Western blot
IF	immunofluorescence
BMD	Becker muscular dystrophy
CPPs	cell-penetrating peptides
FDA	Food and Drug Administration
EMA	European Medicines Agency
EEVs	Endosomal Escape Vehicles
gRNA	guide RNA
MCP	bacteriophage coat protein
snRNA	small nuclear RNA
PK	pharmacokinetics
SCNT	somatic cell nuclear transfer
